# Supplementary material for: A UMLS-based spell checker for natural language processing in vaccine safety
Source: BMC Med Inform Decis Mak. 2007 Feb 12;7:3. doi: 10.1186/1472-6947-7-3 (PMC1805499; doi:10.1186/1472-6947-7-3)
Supplement: Additional file 13 — RAP application directory. Sets up directory for RAP (RDF application for PHP) source code [file 1472-6947-7-3-S13.gz › rap/doc/phpdoc/default/RSS_RES.html]

xml version="1.0" encoding="iso-8859-1"?


Docs For Class RSS\_RES


## Class RSS\_RES

Description

Description |
Methods (details)

Located in /vocabulary/RSS\_RES.php (line 20)

Method Summary

Description |
Methods (details)

void
CHANNEL
()

void
DESCRIPTION
()

void
IMAGE
()

void
ITEM
()

void
ITEMS
()

void
LINK
()

void
NAME
()

void
TEXTINPUT
()

void
TITLE
()

void
URL
()

Methods

Description |
Methods (details)

CHANNEL (line 22)

void

CHANNEL
()

DESCRIPTION (line 70)

void

DESCRIPTION
()

IMAGE (line 28)

void

IMAGE
()

ITEM (line 34)

void

ITEM
()

ITEMS (line 46)

void

ITEMS
()

LINK (line 58)

void

LINK
()

NAME (line 76)

void

NAME
()

TEXTINPUT (line 40)

void

TEXTINPUT
()

TITLE (line 52)

void

TITLE
()

URL (line 64)

void

URL
()

Documentation generated on Fri, 17 Dec 2004 16:18:07 +0100 by phpDocumentor 1.3.0RC3
